# The Composition of Douches

**Published:** 1902-06-07

**Authors:** 


### THE COMPOSITION OF DOUCHES.

Dr. Wyatt Wingrave[1] has made a very useful research into the comparative efficacy of various substances which are commonly employed in the composition of douches, and has arrived at results which are well worth bearing in mind, for although the inquiry related more particularly to Dr. Wingrave's own speciality the principles which he lays down are of general applicability. A douche or irrigant may, he says, be used for the removal of morbid secretions, accumulations, and foreign bodies, for antiseptic purposes or for diagnosis. The solution employed should therefore be, if possible, a solvent of the substance to be removed; it should itself be soluble and should form a clear solution; it should be non-irritating and capable of penetrating the surface tissues; it should be chemically compatible with the most effective antiseptics; and should be both economical and readily available. Most important of all is its power of dissolving the discharge which requires to be removed, for many solutions in common use as douches actually precipitate and harden the substances they are intended to remove. Even water by itself is by no means the most effective irrigant, since of all the organic substances which are likely to require removal albumin is about the only one which it will dissolve. As the outcome of his inquiries Dr. Wingrave gives a series of formulæ. The basis of the majority of them is formed by a solution of from $1\frac{1}{2}$ to 3 drachms of sodium sulphate to 20 fluid ounces of water, that is a solution of from 1 to 2 per cent. This may be used by itself or with additions to each pint of 40 grains of sodium bicarbonate or borax; or, to the weaker solution, 10 grains of chinosol, or 3 fluid drachms of sanitas, or 2 grains of perchloride of mercury, or 2 grains of potassium permanganate may be added. Other useful formulæ are given among which we would specially draw attention to one containing borax 40 grains, sodium bicarbonate 40 grains, glycerine or carbolic acid 45 drops, water to a pint.

[1] Lancet, May 17.

